# Pulsed field ablation of epicardial premature ventricular contractions via a subxiphoid percutaneous approach

**DOI:** 10.1016/j.hrcr.2026.03.019

**Published:** 2026-03-30

**Authors:** Dongdong Que, Liyun Feng, Jing Yan, Yingqi Zhu, Xudong Song, Pingzhen Yang

**Affiliations:** Department of Cardiology, Laboratory of Heart Center, Zhujiang Hospital, Southern Medical University, Guangzhou, China; Heart Center of Zhujiang Hospital, Guangdong Provincial Biomedical Engineering Technology Research Center for Cardiovascular Disease, Guangzhou, China; Heart Center of Zhujiang Hospital, Sino-Japanese Cooperation Platform for Translational Research in Heart Failure, Guangzhou, China

**Keywords:** Pulsed field ablation, Epicardial premature ventricular contractions, Dry epicardial puncture, Subxiphoid percutaneous approach, Coronary spasm

**Key Teaching Points**

• Pulsed field ablation may be a safe and effective option to target epicardial premature ventricular contractions.
• Pulsed field energy delivery near the coronary artery should be performed cautiously.
• Low-dose nitroglycerin administered via coronary infusion, rather than high-dose intravenous injection, may efficiently prevent coronary artery spasm in the absence of hypotension.

## Introduction

Radiofrequency catheter ablation (RFCA) is a well-established treatment of idiopathic outflow tract ventricular arrhythmias (VAs), with high success rates ranging from 80% to 90%.^1^^,^^2^ However, outcomes may be less favorable when VAs originate from epicardial sites because of the technical challenges associated with dry epicardial puncture. Coronary veins have been identified as potential alternatives for mapping and ablating epicardial VAs.^3^ In cases of distinct VAs characterized by an abrupt loss of the R wave in lead V_2_ compared with leads V_1_ and V_3_—referred to as the pattern break in lead V_2_ (PBV2)—which suggests an origin near the anterior interventricular sulcus and adjacent to proximal coronary arteries, RFCA may be limited by catheter accessibility and the risk of injury to adjacent structures.^4^ Herein, we report a case of PBV2-morphology premature ventricular contractions (PVCs) treated with pulsed field ablation (PFA) via a subxiphoid percutaneous approach.

## Case report

**Figure 1:**
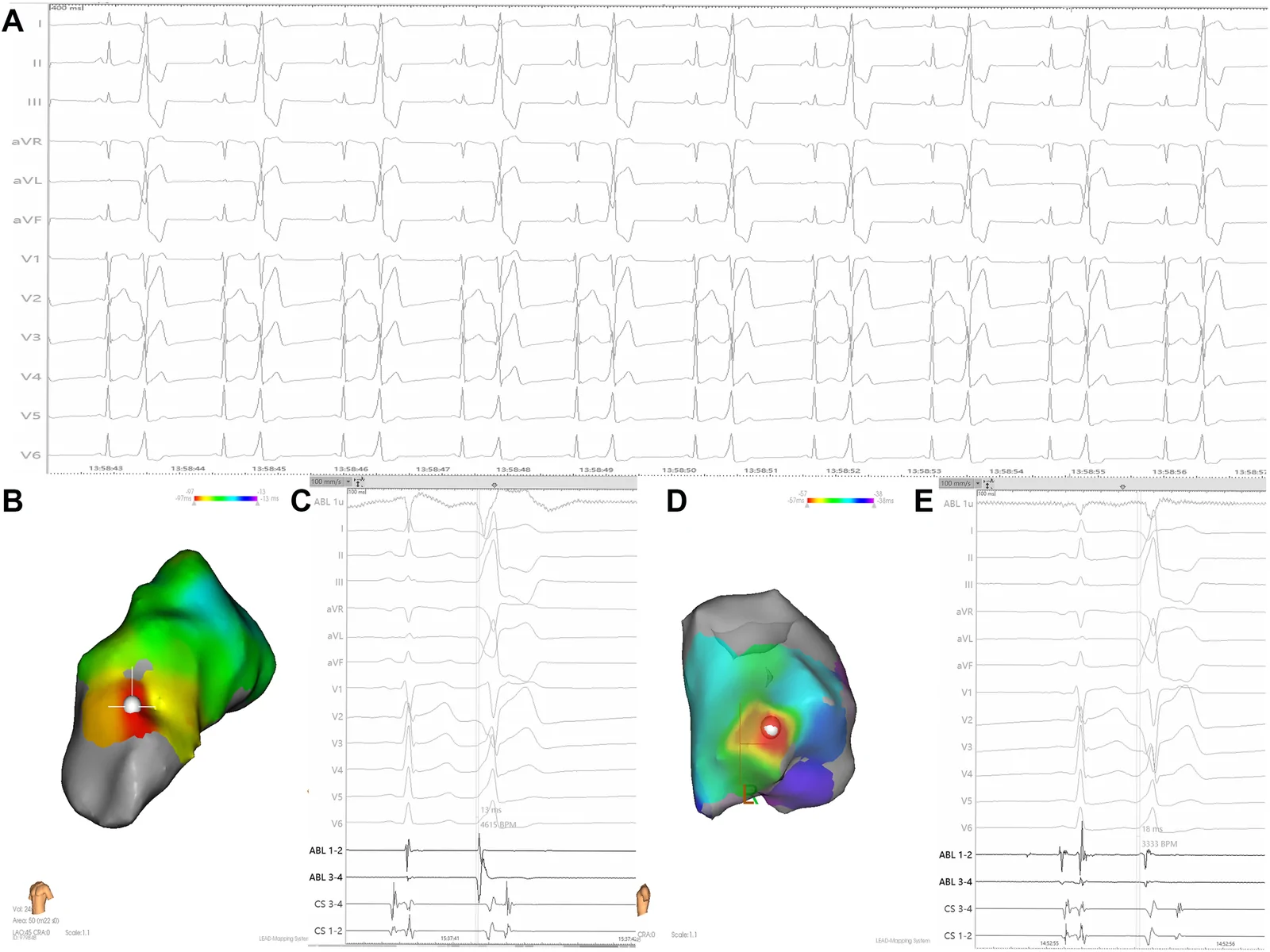
Morphology and activation mapping of premature ventricular contractions (PVCs). **A:** Morphology of PVCs. **B:** Activation mapping in the right ventricular outflow tract, with the *white dot* indicating the relatively earliest activation site (anterior septum. **C:** Comparison of the potential at the *white dot* (from panel B) with that of PVCs. **D:** Activation mapping in the aortic sinus, with the *white dot* indicating the relatively earliest activation site (left coronary cusp). **E:** Comparison of the potential at the *white dot* (from panel D) with that of PVCs.

A 19-year-old man was admitted to our hospital for intolerable palpitations, with a PVC burden of 43% on 24-hour Holter monitoring. Bisoprolol and mexiletine were administered, but only a slight reduction in the number of clinical PVCs was observed. Therefore, electrophysiological intervention was recommended. The PVCs were identified as outflow tract VAs with an idiopathic left bundle branch block morphology (dominant S wave in lead V_1_), a transition zone at lead V_3_, and an abrupt loss of the R wave in lead V_2_ compared to leads V_1_ and V_3_ (PBV2 morphology) (Figure 1A). Echocardiography, chest radiography, and routine biochemical tests yielded negative results, and the patient provided written informed consent before the procedure.

The procedure was performed under conscious sedation using fentanyl infusion. A decapolar catheter was first advanced into the great cardiac vein via the right femoral vein. During PVCs, ventricular activation in the distal coronary sinus (CS12) was not earlier than the body surface QRS complex (Figure 1C). Subsequently, the right ventricular outflow tract was reconstructed 3-dimensionally using a PulsedFA FocalPoint PFA contact catheter guided by a LEAD-Mapping System (Sichuan Jinjiang Electronic Medical Device Technology Co., Ltd., Chengdu, Sichuan, China). The anterior septum exhibited slightly earlier activation (−13 ms) (Figure 1B and 1C); however, the pacing morphology differed. Mapping of the aortic sinus revealed that the earliest activation in the left coronary cusp was nearly identical to that in CS12 (18 ms) (Figure 1D and 1E). An attempted ablation procedure at the right ventricular anterior septum was unsuccessful.

**Figure 2:**
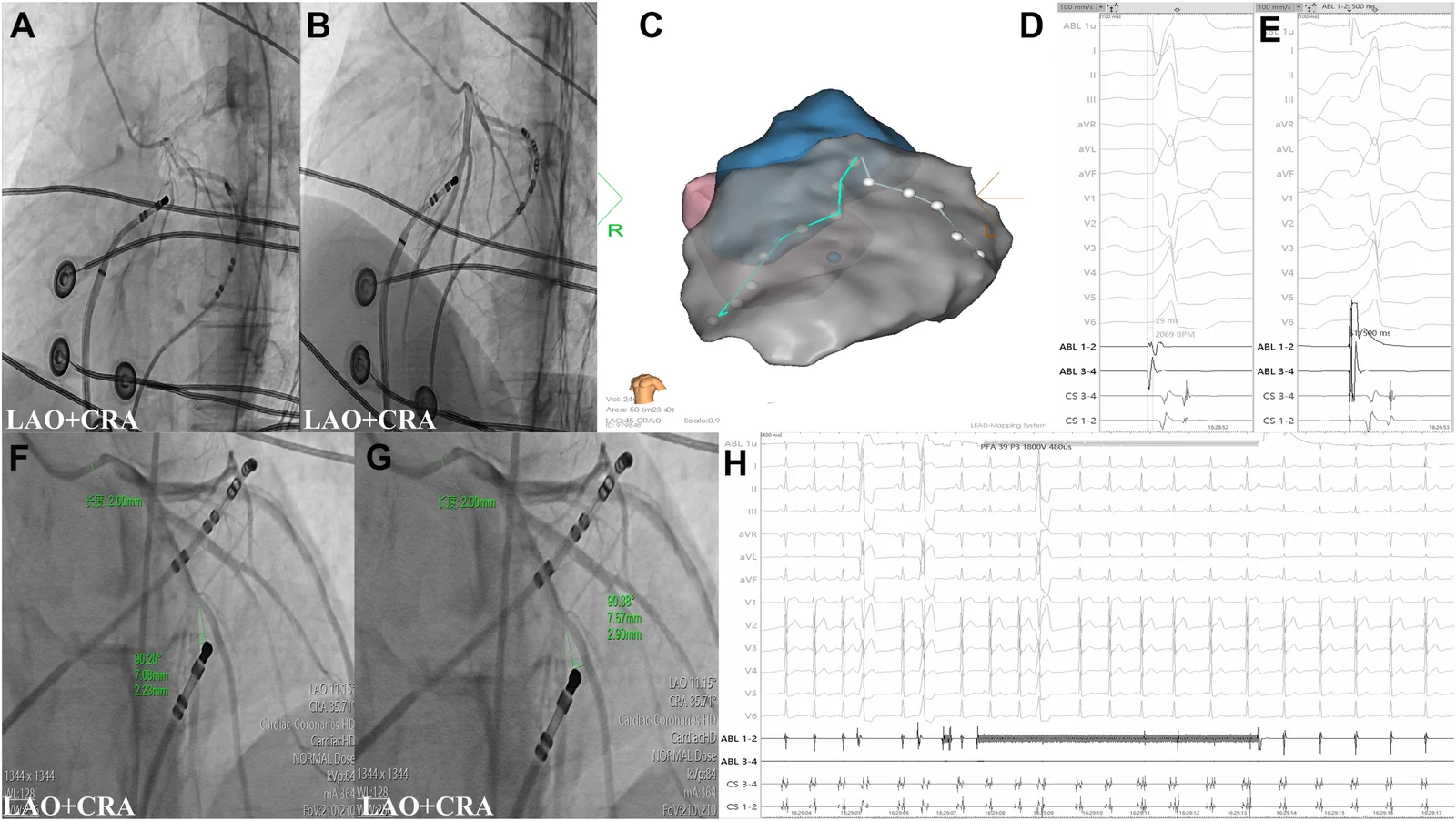
Location of premature ventricular contractions (PVCs) and pulsed field ablation (PFA) energy delivery. **A:** Angiography of the left coronary artery in the left anterior oblique + cranial (LAO + CRA) view before PFA; the PFA catheter was positioned below the bifurcation of the left anterior descending artery (LAD) and the second diagonal branch, where the target site was located. **B:** Angiography of the LAD in the LAO + CRA view after PFA. **C:** 3-dimensional reconstruction of the left ventricular epicardium. The *light blue dotted line* indicates the LAD; the *gray line* indicates the left circumflex artery (LCX); and the *dark blue dot* indicates the target focus. **D:** Comparison of the potential at the *dark blue dot* (from panel C) with that of PVCs. **E:** Pace mapping at the epicardial target site. **F:** Distances between the catheter and the bifurcation (7.68 mm) and LAD (2.23 mm), measured using a 6-F angiographic catheter (2.00 mm) as a reference. **G:** Distances between the catheter and the bifurcation (7.57 mm) and the second diagonal artery (2.90 mm), measured using a 6-F angiographic catheter (2.00 mm) as a reference. **H:** Electrocardiographic documentation of immediate PVC suppression after PFA.

Ultimately, challenging dry epicardial puncture was successfully performed using fluoroscopic guidance in the left lateral 90° view via a subxiphoid percutaneous approach. A PFA catheter was inserted together with a Swartz SL-1 sheath (Abbott, Chicago, IL) over the guidewire and subsequently used for epicardial mapping. The earliest activation time (−29 ms) was localized to the middle portion of the left ventricle, and the pacing morphology matched that of clinical PVCs (Figure 2C–2E). The target focus was confirmed below the bifurcation of the left anterior descending artery (LAD) and the second diagonal branch, with an extremely close distance to the LAD as assessed by angiography (Figure 2A). The distances were approximately 7.63 mm (average of 7.57 and 7.68 mm) below the bifurcation, approximately 2.23 mm from the LAD, and approximately 2.99 mm from the second diagonal branch, measured with reference to a 6-F angiographic catheter in the left anterior oblique and cranial views (Figure 2F and 2G). To prevent vasospasm, 300 μg of nitroglycerin was infused into the left coronary artery via the angiographic catheter before PFA energy delivery. Medium-level energy (P3/1800 V/480 μs) was initially applied, resulting in an immediate elimination of frequent PVCs (Figure 2H). With preinfusion of fentanyl citrate and the use of bipolar PFA energy delivery, the patient experienced no chest pain and showed no surface electrocardiographic changes. 2 additional medium-level energy applications were delivered for consolidation. Subsequent angiography revealed no evidence of LAD spasm (Figure 2B). Repeated bolus infusions of high-dose isoproterenol (15 μg) failed to provoke PVCs during the 30-minute observation period after PFA. A pigtail catheter was temporarily left in the epicardial space for drainage and was removed the following day. The patient remained asymptomatic without palpitations, and Holter monitoring confirmed the absence of PVCs postprocedure as well as at 6-month outpatient follow-up.

## Discussion

As an ultrashort, high-frequency, and nonthermal technique, PFA has recently emerged as an optimal alternative to RFCA for atrial fibrillation.^4^ In addition, it has been attempted for the treatment of PVCs in several individual cases with favorable results, particularly in those refractory to conventional therapies.^5^ Mestrovic et al^6^ reported a case of pseudoepicardial monomorphic PVCs targeted at the distal segment of the coronary venous system near the mitral valve, where PFA achieved complete suppression after temporary suppression with RFCA. To our knowledge, we may be the first to report the application of PFA energy for true epicardial PVCs via a subxiphoid percutaneous approach.

Hayashi et al^7^ abandoned RFCA in 2 patients with PVCs exhibiting a PBV2 morphology because the earliest activation site was located in the epicardium adjacent to the LAD. RFCA causes thermal injury to the myocardium, which may extend to adjacent structures such as coronary vessels, whereas PFA offers tissue selectivity without thermal conduction, despite the potential risk of vascular spasm; therefore, given these technical challenges, a PFA system and tools for dry epicardial puncture were prepared preprocedurally in our case on the basis of the surface electrocardiographic pattern consistent with the PBV2 morphology. In our patient, the epicardial target showed significantly earlier activation than the sinus aorta, great cardiac vein, and right ventricular outflow tract. Despite the close proximity to the LAD, PFA was delivered in a stepwise manner and proved safe and effective with prophylactic intracoronary infusion of low-dose nitroglycerin. This finding highlights the potential advantage of PFA over RFCA in managing PVCs with epicardial targets adjacent to critical coronary vessels, as it avoids the thermal injury–related risks of RFCA while addressing the concern of vascular spasm through appropriate prophylactic measures.

Hemolysis, pericardial effusion, and vasovagal reactions are the most commonly reported energy-specific adverse events associated with PFA.^8^ However, coronary artery spasm may represent a prominent potential complication of epicardial PFA, with an incidence ranging from 0.08% to 0.14% in patients undergoing atrial fibrillation ablation.^9^ The underlying mechanism is likely a cascade of local inflammatory responses in vascular smooth muscle directly triggered by pulsed electric fields. Gunawardene et al^10^ reported a case of severe clinically delayed coronary spasm during cavotricuspid isthmus ablation using a pentaspline PFA catheter, despite prophylactic high-dose intravenous nitroglycerin before the procedure. Pulsed electric fields induce a transient cellular electroshock effect, further prolonging the metabolic clearance of hyperphosphorylated myosin light chains while reducing the sensitivity of vascular smooth muscle to nitric oxide.^8^ Accordingly, administration of higher doses of nitroglycerin via superficial veins has been recommended for prophylaxis. Nevertheless, extensive intravenous nitroglycerin administration increases the risk of hypotension. Furthermore, Tam et al^11^ observed mild coronary artery narrowing on optical coherence tomography at 3-month follow-up, supporting that heightened vigilance and long-term monitoring remain necessary.

Coronary angiography was performed to confirm the anatomical relationship between the target focus and the LAD before PFA. Subsequently, low-dose nitroglycerin was infused directly into the left coronary artery via the angiographic catheter. PFA was then delivered immediately, while the angiographic catheter was kept at the coronary ostium. Postprocedural angiography was performed afterward, revealing persistent significant dilation of the LAD. The patient remained asymptomatic without chest pain or electrocardiographic changes during the subsequent observation period. Intracoronary infusion of low-dose nitroglycerin may effectively prevent coronary spasm, even when PFA energy is delivered adjacent to the coronary artery.

## Conclusion

Epicardial PFA can be a safe and effective option to target epicardial PVCs. Compared with high-dose intravenous nitroglycerin administration, low-dose nitroglycerin infused via the coronary artery effectively prevented coronary artery spasm in this case, regardless of the presence of hypotension. However, additional clinical observations are required to validate this finding.

## Disclosures

The authors have no conflicts of interest to disclose.
